# Supplementary material for: Two-gigapascal-strong ductile soft magnets
Source: Nat Commun. 2024 Nov 22;15:10119. doi: 10.1038/s41467-024-53793-2 (PMC11584709; doi:10.1038/s41467-024-53793-2)
Supplement: Supplementary file 1 — Supplementary Information [file 41467_2024_53793_MOESM1_ESM.pdf]

Supplementary Information

Two-gigapascal-strong ductile soft magnets

Liuliu Han[1], Nicolas J. Peter[2], Fernando Maccari[3], András Kovács[4], Jin Wang[2], Yixuan Zhang[3], Ruiwen Xie[3], Yuxiang Wu[1], Ruth Schwaiger[2], Hongbin Zhang[3], Zhiming Li[5], Oliver Gutfleisch[3], Rafal E. Dunin-Borkowski[4], Dierk Raabe[1]

[1]Max Planck Institute for Sustainable Materials, Max-Planck-Straße 1, 40237 Düsseldorf, Germany
[2]Institute of Energy and Climate Research (IEK-2), Forschungszentrum Jülich, 52425 Jülich, Germany
[3]Institute of Materials Science, Technical University of Darmstadt, 64287 Darmstadt, Germany
[4]Ernst Ruska-Centre for Microscopy and Spectroscopy with Electrons and Peter Grünberg Institute, Forschungszentrum Jülich, 52425 Jülich, Germany
[5]School of Materials Science and Engineering, Central South University, 410083 Changsha, China

Correspondence to Dr. Liuliu HAN l.han@mpie.de (L.H.)

Supplementary Figures

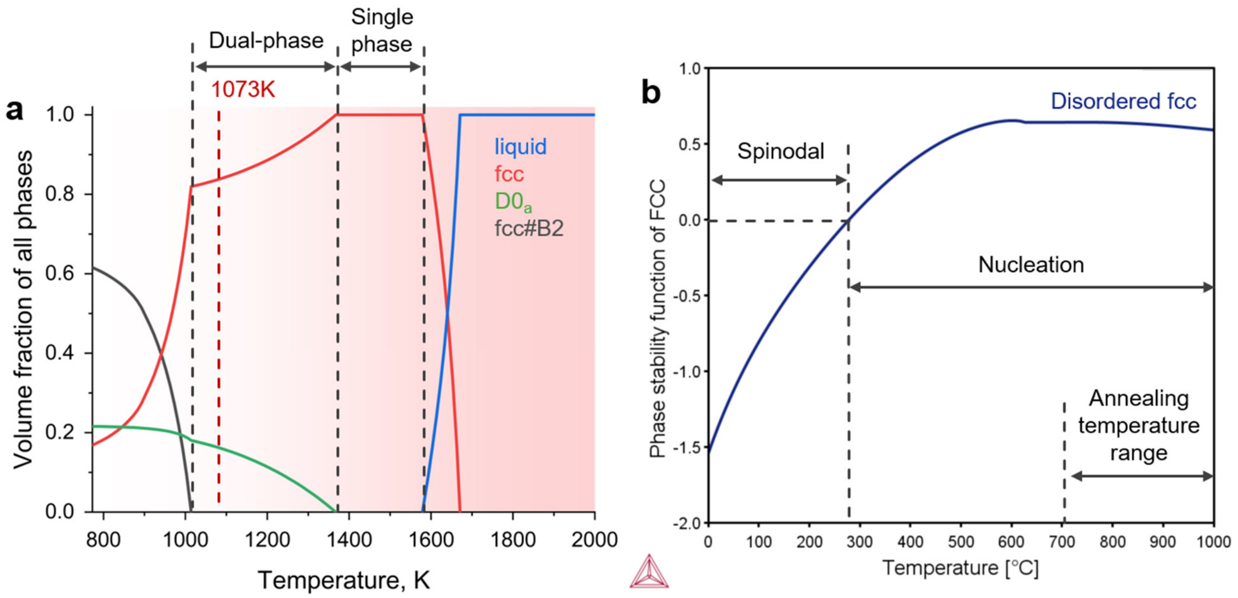

Supplementary Figure 1. Thermodynamic analysis of the $Fe_{35}Co_{30}Ni_{30}Ta_5$ (at.%) alloy calculated using Thermo-Calc software.

a Calculated equilibrium phase diagram for the $Fe_{35}Co_{30}Ni_{30}Ta_5$ (at.%) alloy system, showing that it has a solid solution single-phase structure at higher temperature (1365 K~1670 K). A second phase of different crystal structures is formed at lower temperatures (1015 K~1365 K). The $D0_a$ phase is regarded as an ordered structure with low crystal symmetry based on a hexagonal crystal lattice, i.e., the $D0_{19}$ crystal structure. **b** The stability function of the disordered fcc phase is plotted as a function of temperature, showing the local curvature of the free energy curve. The system assumes a spinodal reaction below 550 K (negative value) and a nucleation reaction above 550 K (positive value).

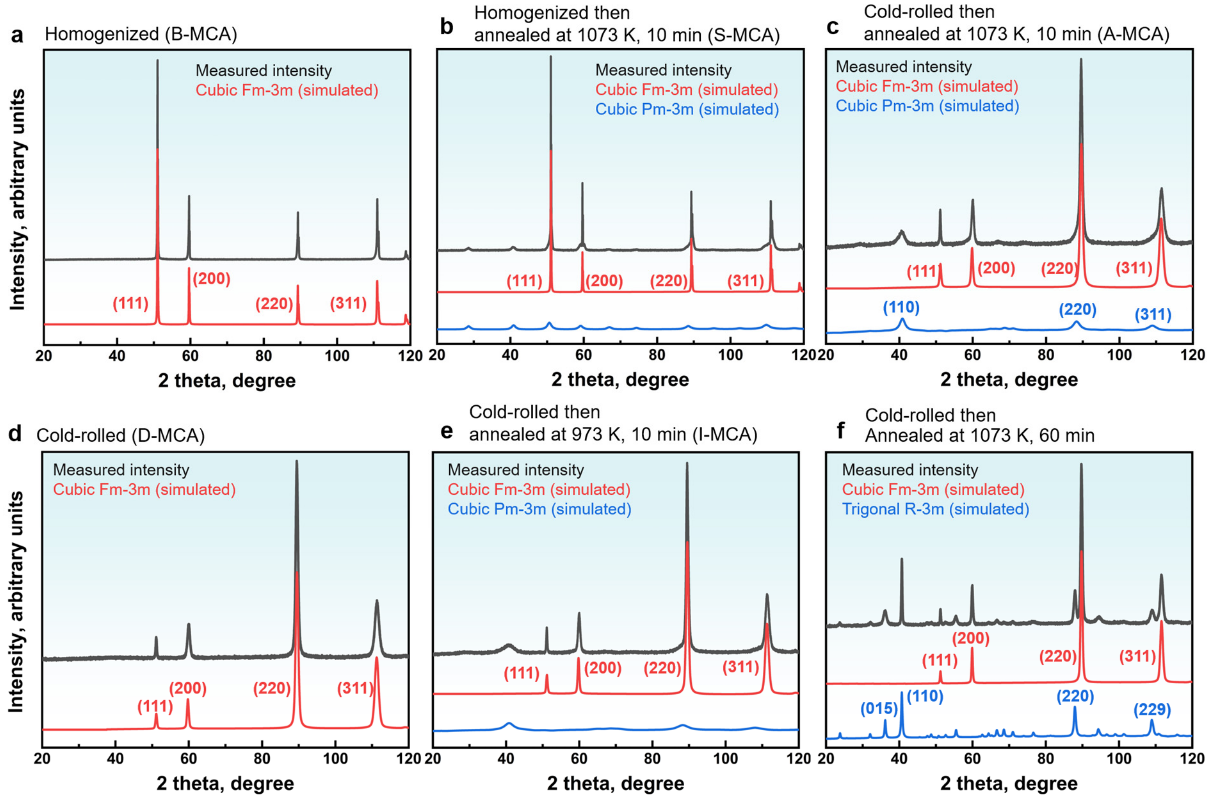

Supplementary Figure 2. X-ray diffraction (XRD) analysis.

Measured and simulated XRD patterns showing the phase structure and texture of the investigated $Fe_{35}Co_{30}Ni_{30}Ta_5$ (at.%) multicomponent materials obtained by different thermomechanical processing. **a** B-MCA. **b** S-MCA. **c** A-MCA. **d** D-MCA. **e** I-MCA. **f** 1073 K, 60 min-MCA. No diffraction peaks of ordered hcp are detected, indicating that the $L1_2$ phase constitutes the major fraction of the nanoprecipitates in the A-MCA.

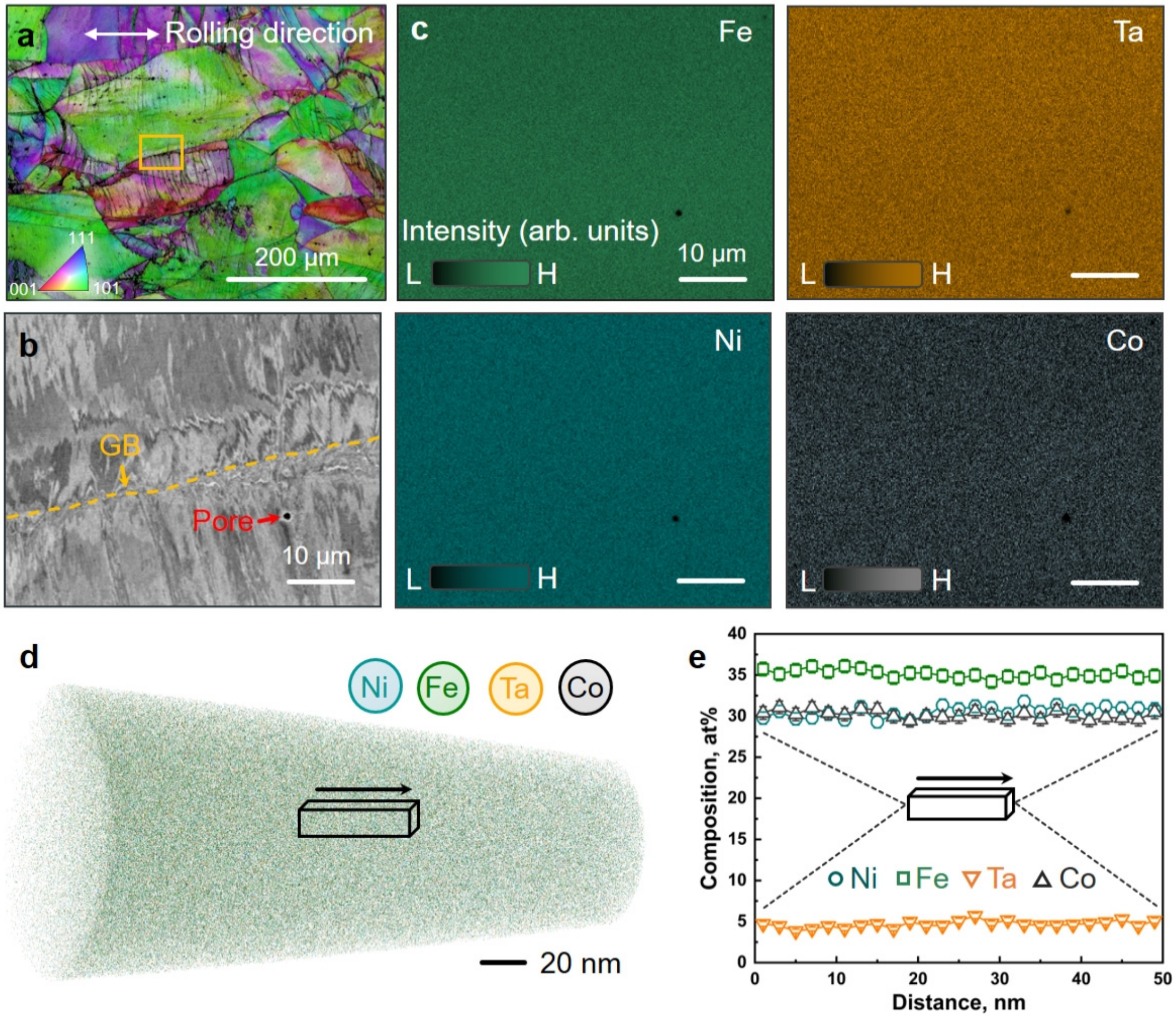

Supplementary Figure 3. Microstructure of the D-MCA material from the micro- to the near-atomic scale.

a EBSD-IPF map of the cold-rolled microstructure. The average grain size is 91±45 μm. **b** SEM image corresponding to the region marked by an orange solid frame in (**a**). The grain boundary (GB) is highlighted by an orange dashed line. A pore is marked by a red arrow. **c** EDS maps of all constituent elements in the sample region shown in (**b**). L, low intensity; H, high intensity. The average composition measured using EDS mapping is $Fe_{26.7}Co_{28.7}Ni_{28.3}Ta_{13.3}$ (at.%). **d** 3D reconstruction map of an APT tip, showing a uniform elemental distribution at the near-atomic scale. **e** Chemical profiles in the marked black region (50 nm×10 nm×10 nm) along the arrow in (**d**). The error bars are estimated as described in Methods.

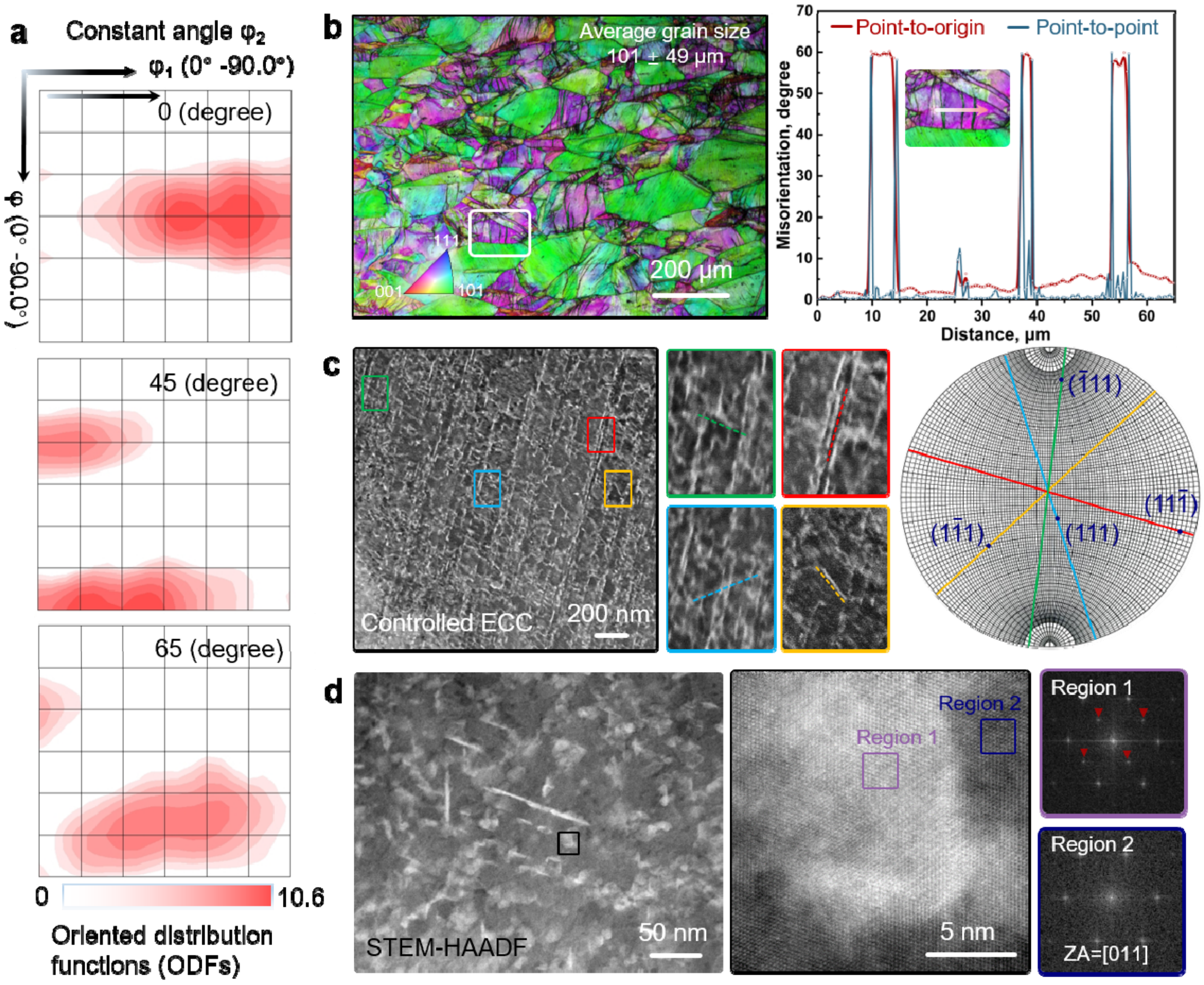

Supplementary Figure 4. Microstructure of the A-MCA material from the micro- to the atomic scale.

a EBSD-oriented distribution functions maps showing an {011}<211> texture[1] **b** EBSD-IPF map and corresponding misorientation profile recorded along the arrow in the region marked by a white frame, showing cold-rolling-induced deformation twinning. **c** Control-ECC analysis, showing that the longitudinal edges of the precipitates are crystallographically parallel to the $[110]_{fcc}$ direction. **d** Overview STEM HAADF image (left), enlarged view (middle, corresponding to the region marked by a black solid frame), and corresponding FFT patterns (right), showing that a transversal cross-section of the precipitates (ordered, region 1, purple) is coherent with the fcc matrix (disordered, region 2, navy).

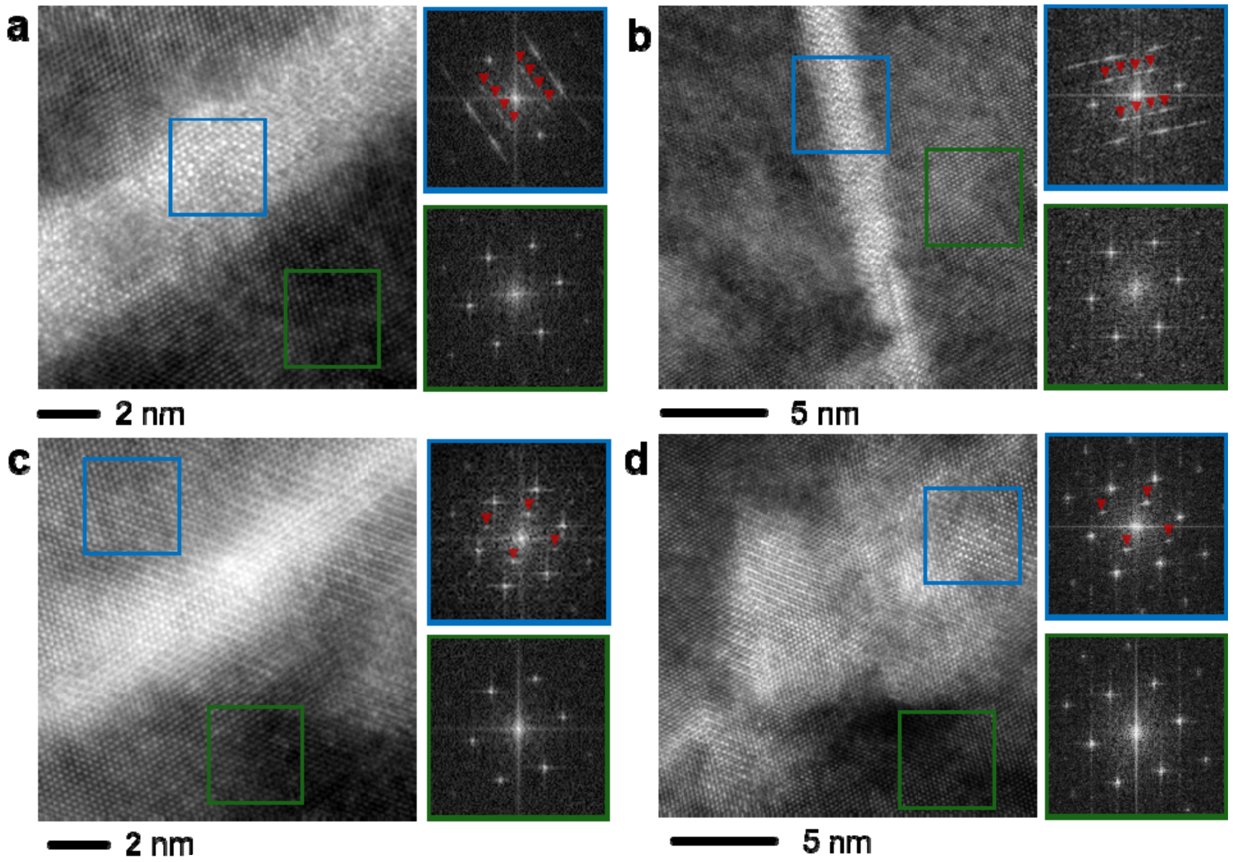

Supplementary Figure 5. Atomically-resolved STEM HAADF micrographs and FFT patterns recorded from the plate-like precipitates in the A-MCA material.

a, b Representative regions showing that the hcp crystal structure precipitates (blue regions) are coherent with the disordered matrix (green regions). **c**, **d** Representative regions showing that the $L1_2$ crystal structure precipitates (blue regions) are coherent with the disordered matrix (green regions).

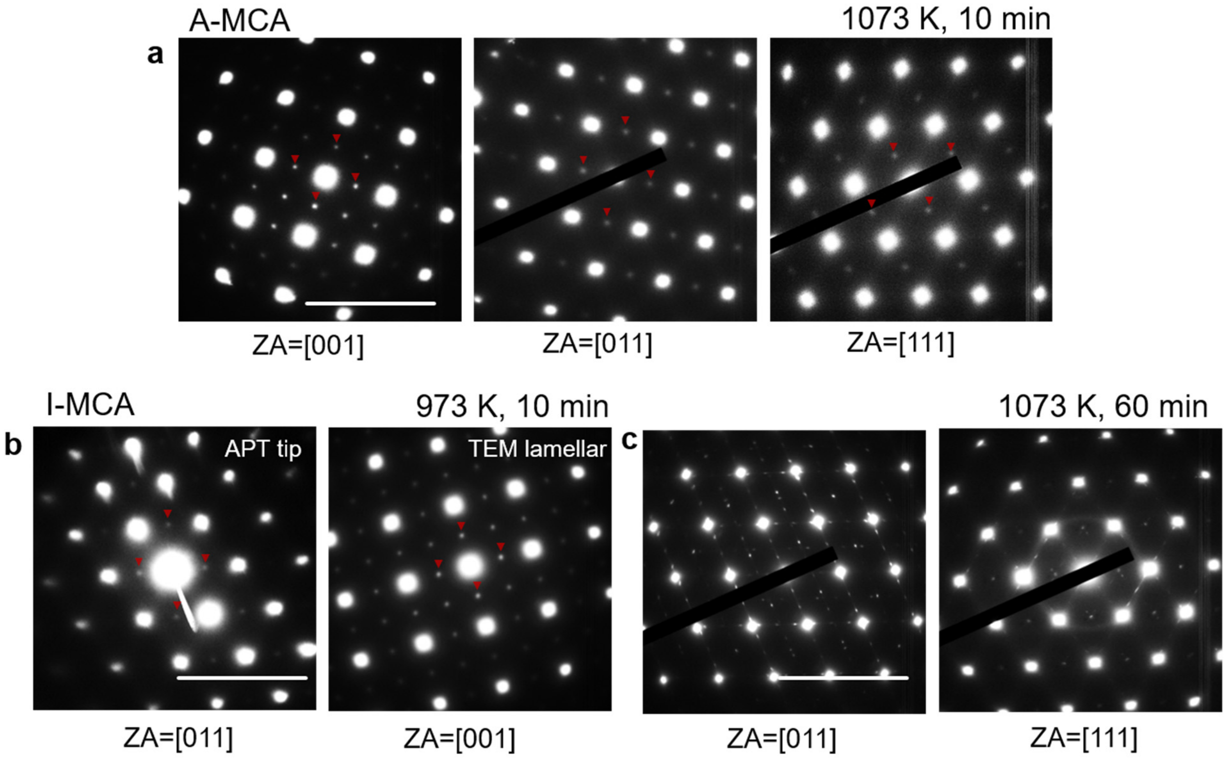

Supplementary Figure 6. Selected area electron diffraction (SAED) analysis.

a SAED patterns recorded from the A-MCA material at [001], [011] and [111] zone axis (ZA) orientations from a TEM lamella. **b** SAED patterns recorded the I-MCA material at the [011] ZA from the correlative APT-TEM specimen (left) and at the [001] ZA from a TEM lamella. Pronounced superlattice diffraction spots are observed for the I-MCA material, indicating that the solid-state phase transformation during annealing is thermodynamically the same as in A-MCA. **c** SAED patterns recorded from the 1073 K, 60 min annealed MCA material at the [011] and [111] ZAs from the TEM lamella specimen. Scale bar, 1/(0.086 nm).

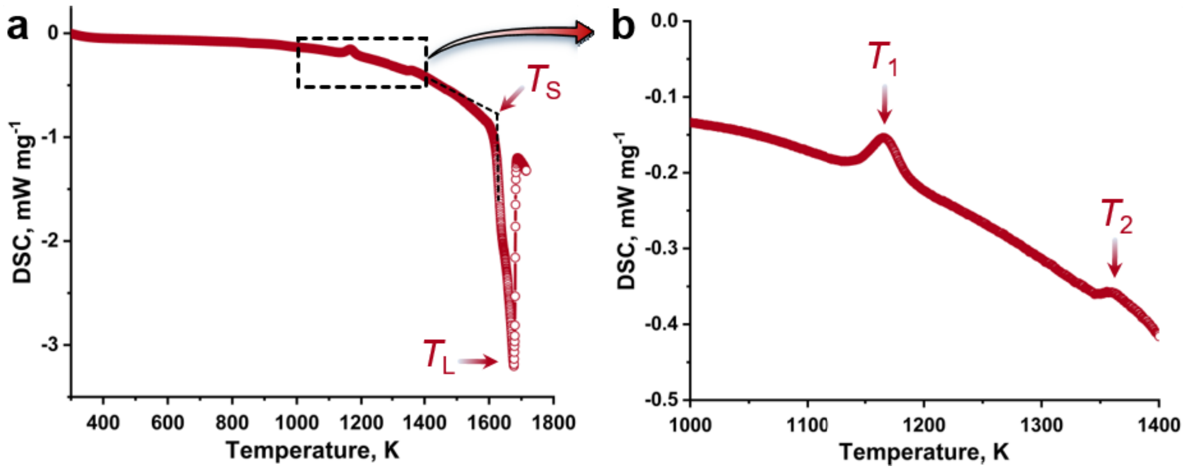

Supplementary Figure 7. Differential scanning calorimetry (DSC) analysis of the $Fe_{35}Co_{30}Ni_{30}Ta_5$ (at.%) multicomponent material.

a DSC analysis during heating showing the solidus (T_S) and liquidus (T_L) melting temperatures are 1620 K and 1680 K, respectively. **b** Enlarged view identical to the black dashed frame in (**a**) showing an exothermic effect (T_1) at 1141 K, indicating that the original sample was not in equilibrium but metastable. A second-order effect (T_2) is observed at 1348 K, indicating the end-of-phase transition reaction.

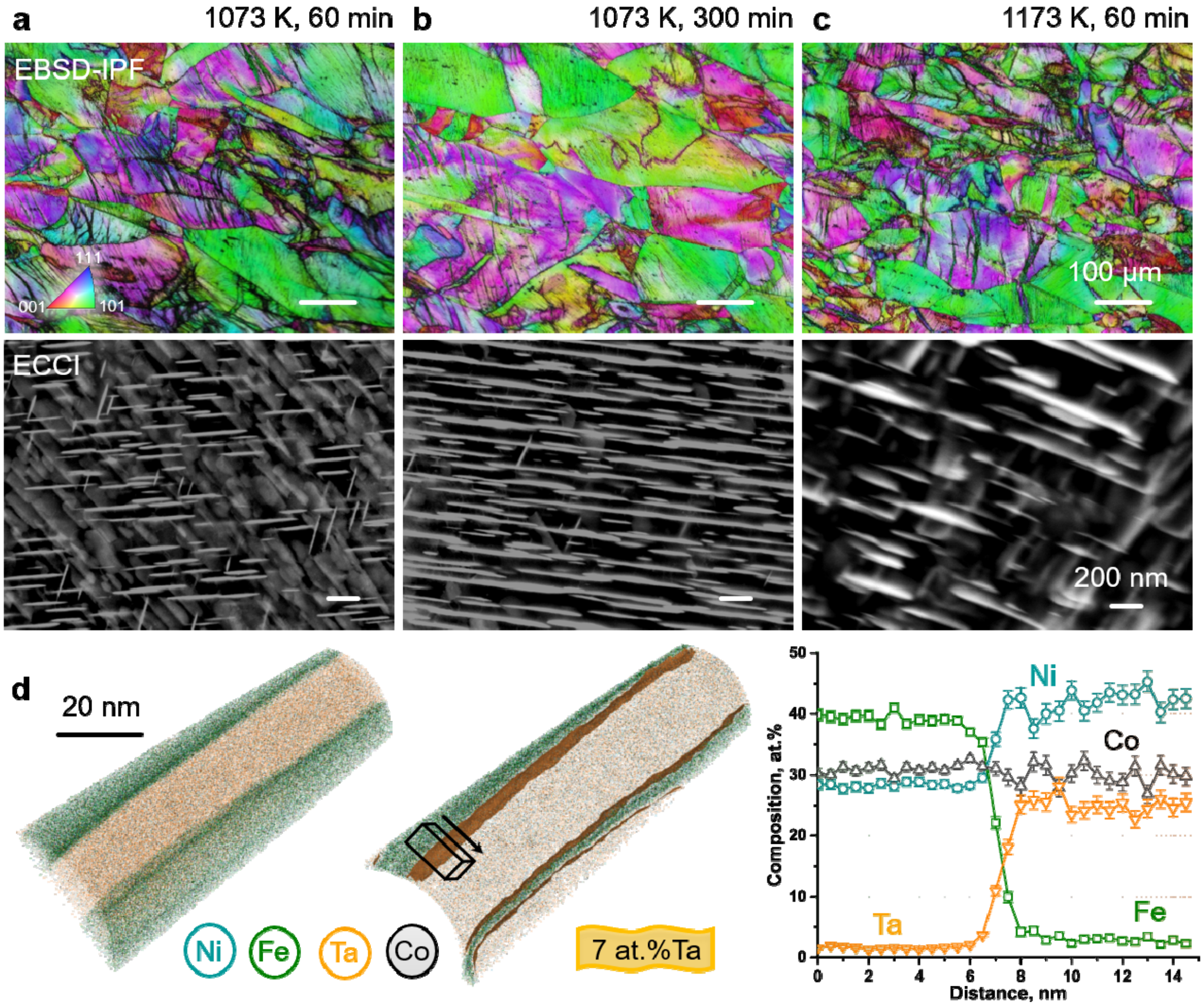

Supplementary Figure 8. Microstructure analysis of the MCAs through further isothermal heat treatment (60~300 min at 1073~1173 K) of the cold-rolled D-MCA.

EBSD-IPF maps (top images) showing the structure containing deformation-induced texture. No recrystallization was observed. The ECC images (middle images) showing the morphology of the plate-shaped precipitates. **a** MCA annealed at 1073 K for 60 min. **b** MCA annealed at 1073 K for 300 min. **c** MCA annealed at 1173 K for 60 min. **d** 3D reconstruction map of the MCA annealed at 1073 K for 60 min (left), and a 10 nm-thick slice (middle) highlighted by 7 at.% Ta isosurface. The corresponding chemical profiles (right) of the black rectangular (15 nm×10 nm×10 nm) along the arrow showing the compositions of the fcc matrix and precipitate are $Fe_{39.3}Co_{30.8}Ni_{28.3}Ta_{1.6}$ and $Ni_{42.6}Co_{30.0}Ta_{24.6}Fe_{2.8}$ (at.%), respectively. The error bars are estimated as described in Methods.

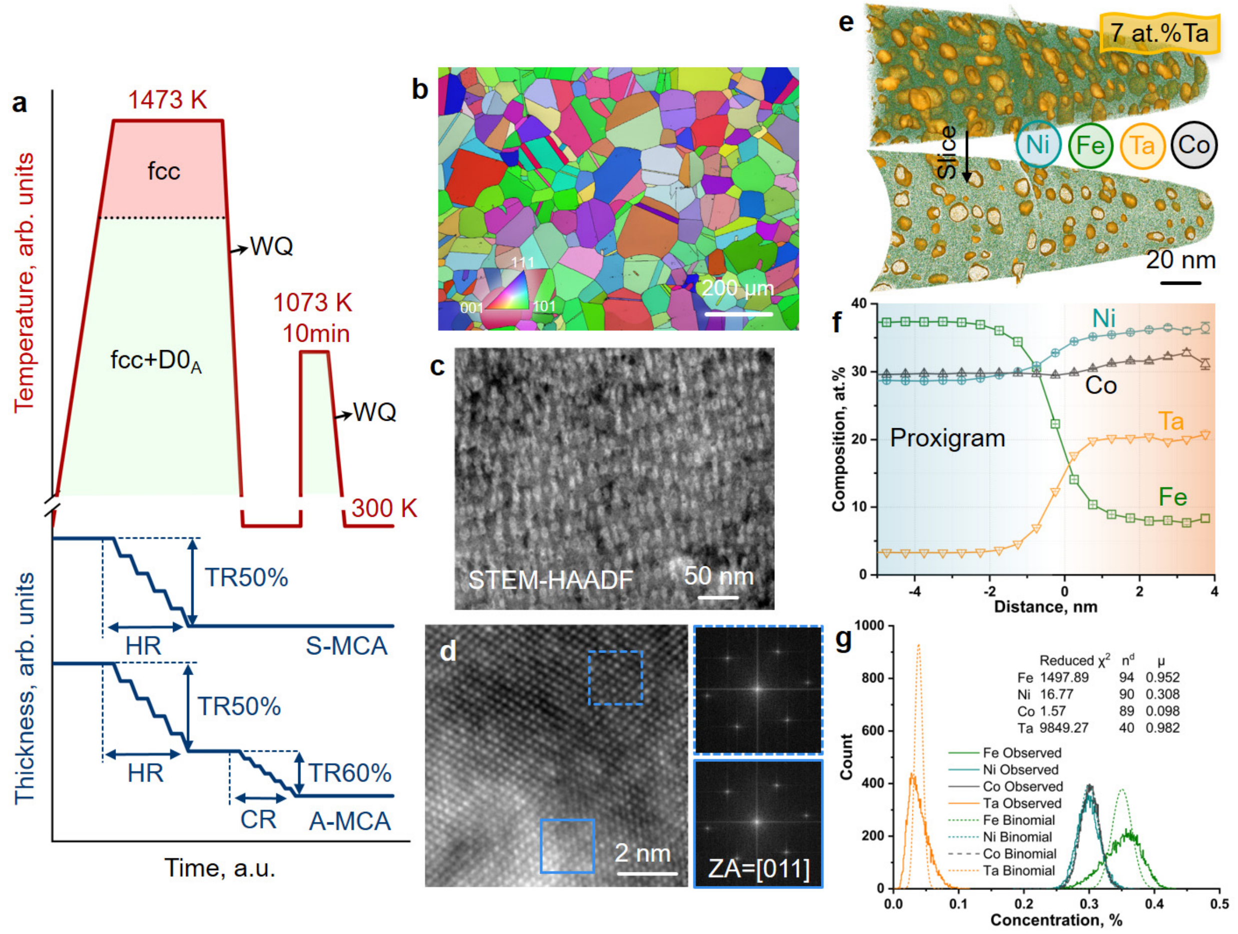

Supplementary Figure 9. Microstructure of the S-MCA material from the micro- to the near-atomic scale.

a Schematic illustration of the thermomechanical processing of the S-MCA and A-MCA materials. **b** EBSD-IPF map showing the equiaxed grain structure. **c** STEM HAADF micrograph showing the high-density spherical-shaped precipitates. **d** Enlarged atomically-resolved micrograph and FFT patterns of representative low Z contrast (fcc matrix, dashed blue frame) and high Z contrast (precipitate, solid blue frame) regions, showing that the precipitates are disordered and coherent with the matrix. **e** 3D reconstruction map (top) and a 10-nm-thick slice (bottom) containing a 7 at.% Ta isosurface. **f** Proximity diagram calculated using a 7 at.% Ta isosurface. The fcc matrix and precipitate compositions are $Fe_{37.7}Co_{29.0}Ni_{30.0}Ta_{3.3}$ and $Ni_{37.5}Co_{33.0}Ta_{21.0}Fe_{8.5}$ (at.%), respectively. The error bars are estimated as described in Methods. **g** Statistical frequency distribution analysis, showing that the experimentally observed binomial curves for Fe and Ta deviate significantly from the binomial simulation, suggesting a strong tendency for elemental

segregation at the near-atomic scale. This is further confirmed by the values of the Pearson coefficient parameter (μ).

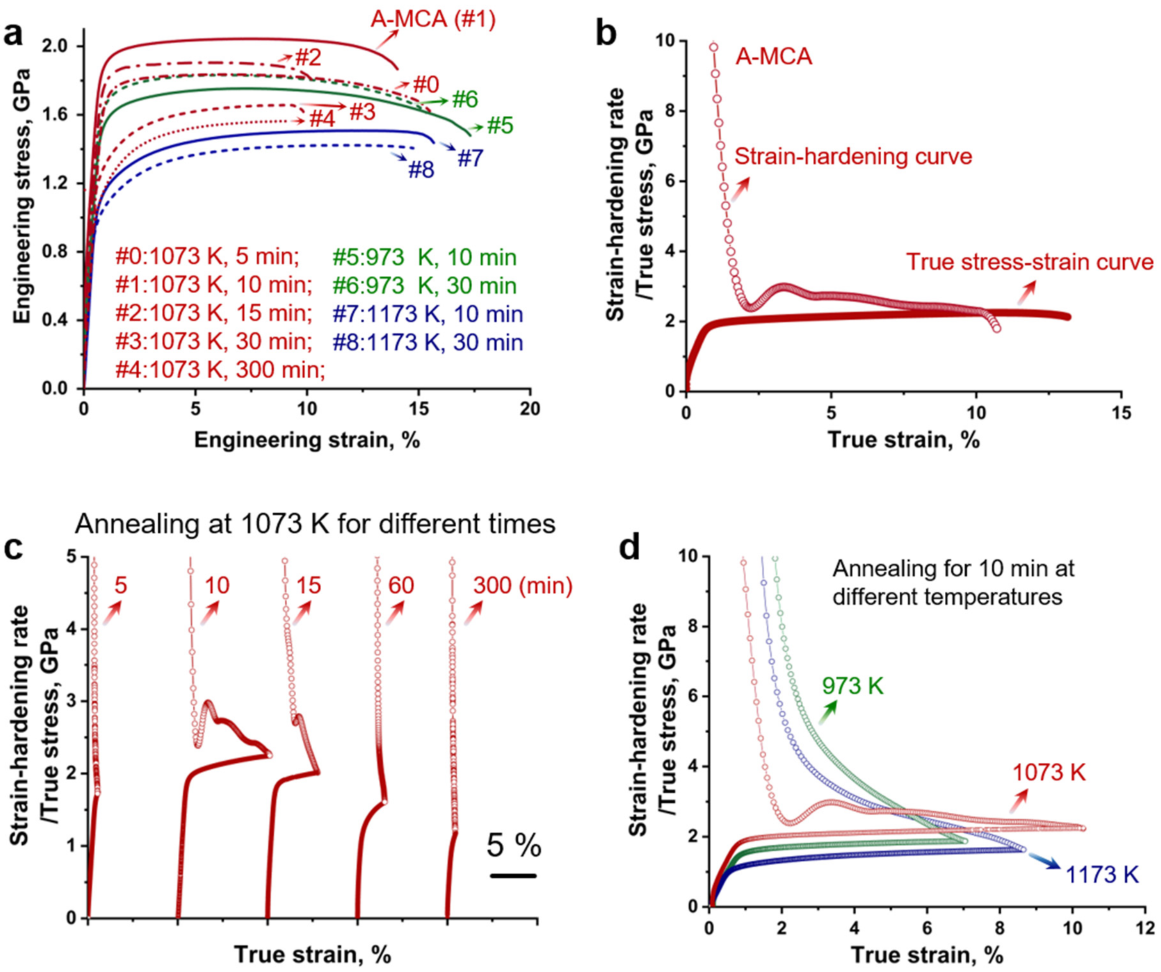

Supplementary Figure 10. Mechanical tensile performance of the investigated $Fe_{35}Co_{30}Ni_{30}Ta_5$ (at.%) multicomponent materials measured at room temperature.

a Representative engineering stress-strain curves. The inset shows the isothermal heat treatment conditions (5~300 min at 973~1173 K). **b** True stress-true strain and strain-hardening-true strain curves of the A-MCA material, showing multi-stage strain-hardening behaviour. For comparison, true stress-true strain and strain-hardening-true strain curves of MCAs containing different sizes, morphologies, structures and compositions of precipitates by isothermal heat treatment are shown in **c** for the same annealing temperature (1073 K) and different times (5~300 min), and **d** the same annealing time (10 min) at different temperatures (973 and 1173 K) as for the A-MCA material.

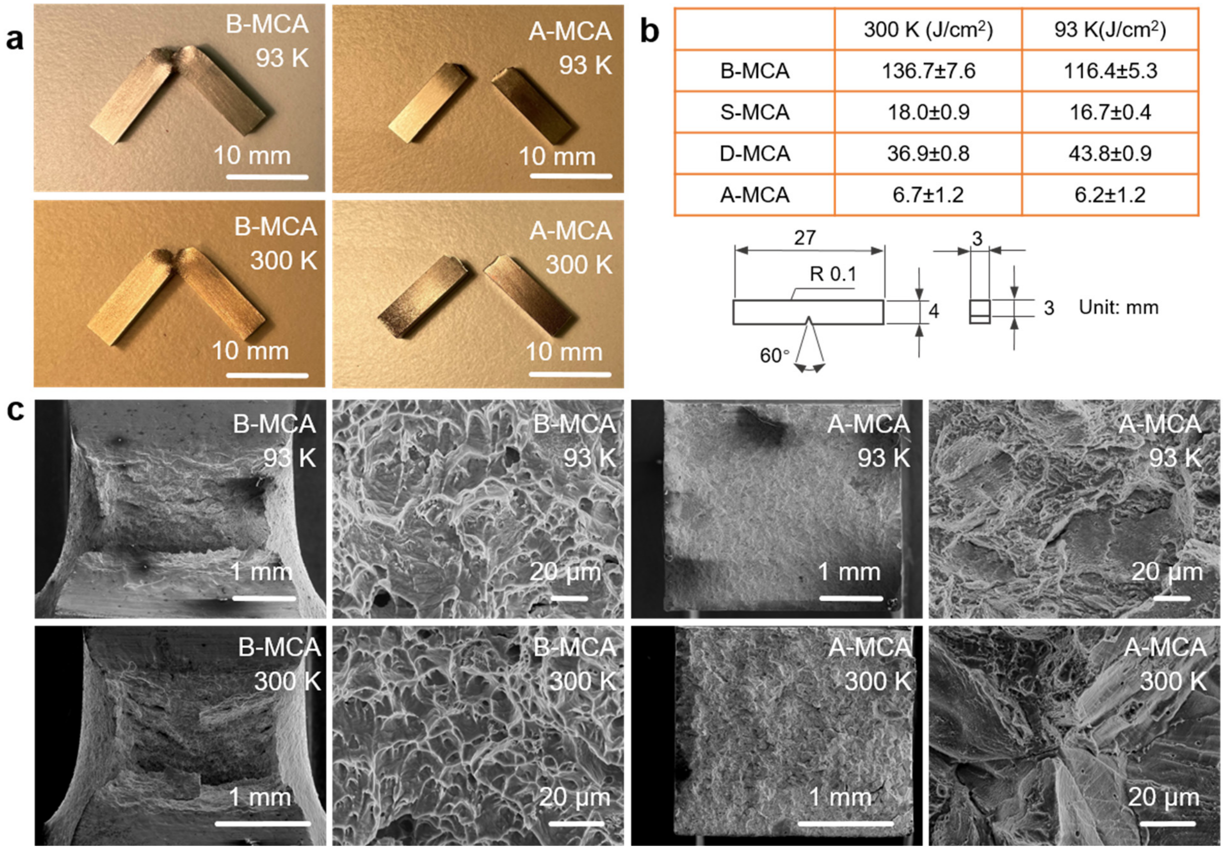

	300 K (J/cm²)	93 K(J/cm²)
B-MCA	136.7±7.6	116.4±5.3
S-MCA	18.0±0.9	16.7±0.4
D-MCA	36.9±0.8	43.8±0.9
A-MCA	6.7±1.2	6.2±1.2

Supplementary Figure 11. Impact toughness performance of the investigated $Fe_{35}Co_{30}Ni_{30}Ta_5$ (at.%) multicomponent materials at room and low temperatures.

a Macroscopic images of the samples after Charpy experiments. **b** Impact energies at room temperature (300 K) and low temperature (93 K), and geometry of the subsize precracked (V-notch) Charpy specimens. All standard deviations are obtained by at least three measurements. The Charpy specimens were machined along the rolling direction. **c** SEM fractography recorded from the center regions of the fracture surfaces.

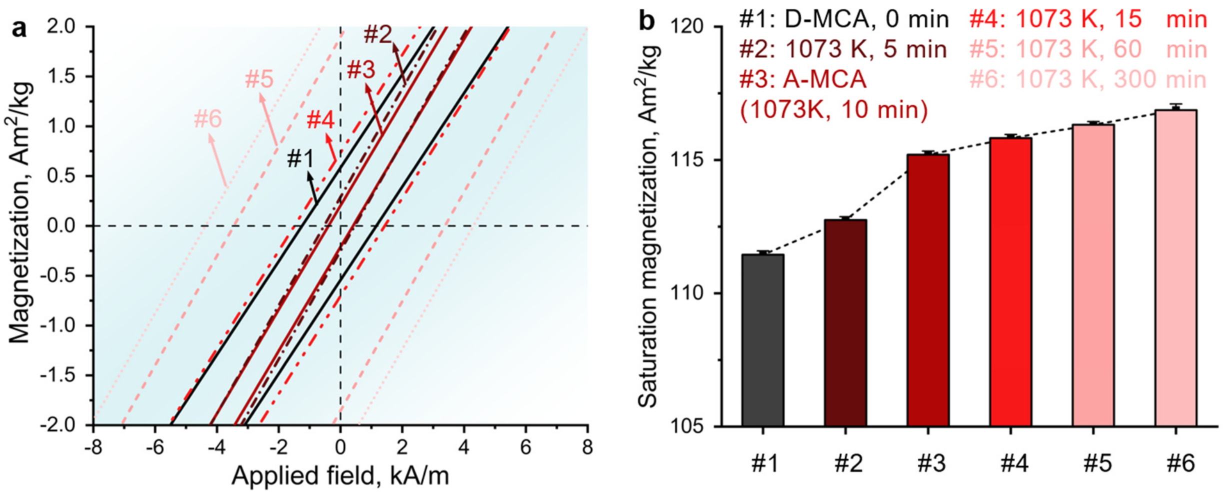

Supplementary Figure 12. Room temperature magnetic performance of the investigated $Fe_{35}Co_{30}Ni_{30}Ta_5$ (at.%) multicomponent materials with different isothermal heat treatments. **a** Enlarged hysteresis loops, showing H_c values. H_c first decreases from 1.17 kA/m (0 min) to 0.36 kA/m (10 min). It then increases to 4.35 kA/m (300 min) with prolonged annealing time at 1073 K. **b** Plot showing M_s of the measured materials. M_s increases slightly on extending the annealing time at 1073 K from 111.5 Am^2 kg^{-1} (0 min) to 116.9 Am^2 kg^{-1} (300 min). All standard deviations are obtained by at least three measurements.

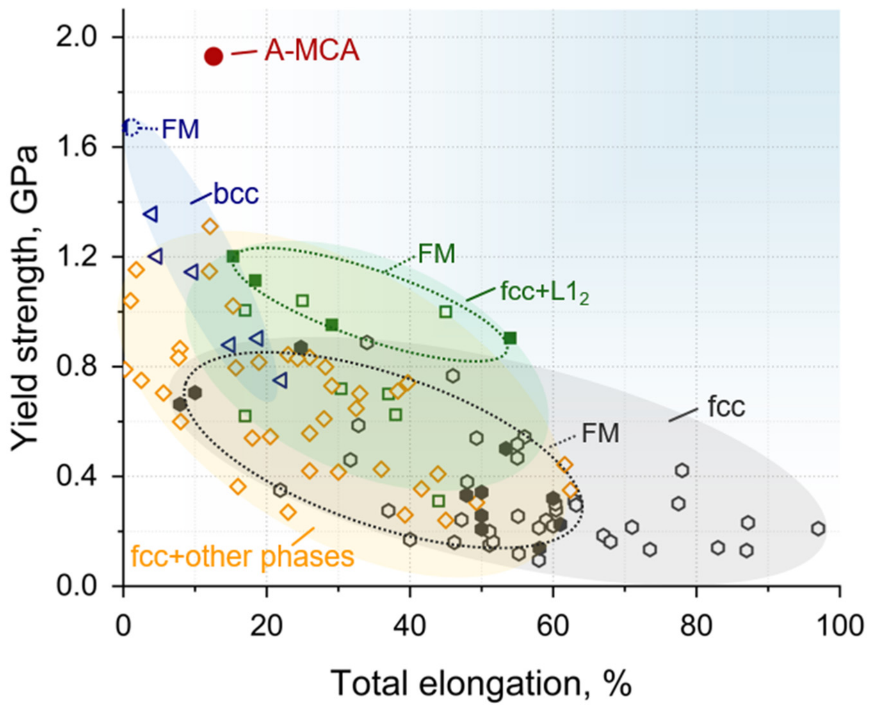

Supplementary Figure 13. Ashby map showing the room temperature tensile yield strength and elongation at fracture of the current A-MCA material and established MCAs[2]

fcc MCAs in the single face-centred cubic structure; bcc MCAs in the single body-centred cubic structure; fcc+L1$_2$, fcc-based MCAs with L1$_2$ precipitation; fcc+other phases, fcc-based MCAs with other phases except for L1$_2$. Dashed regions with FM indicate ferromagnetic MCAs.

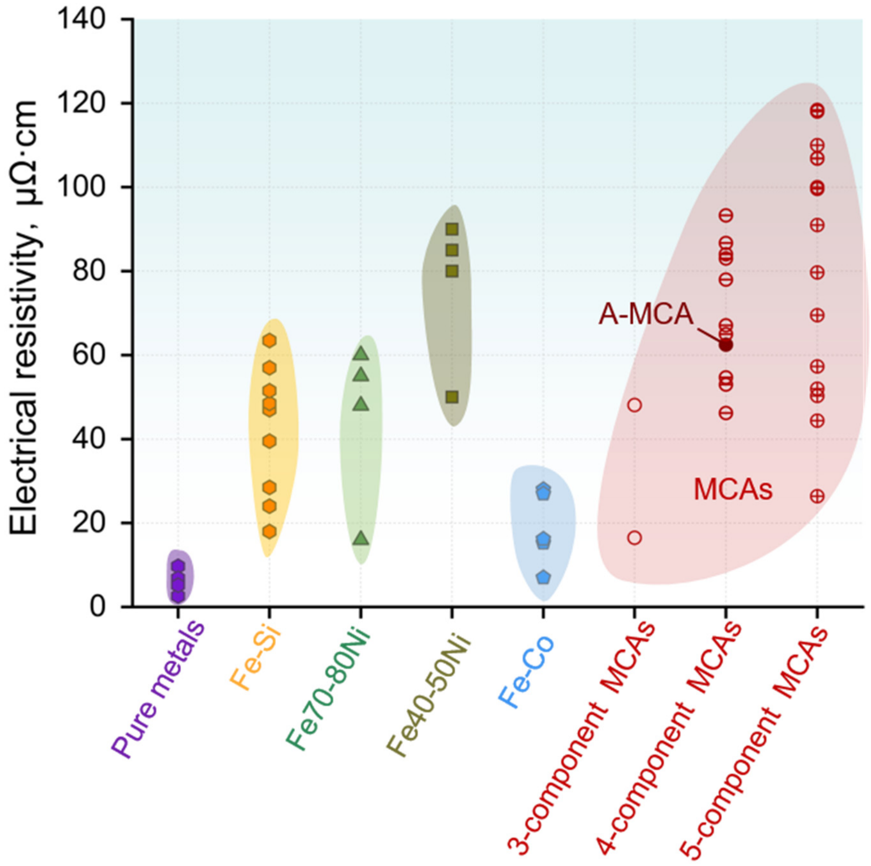

Supplementary Figure 14. Electrical resistivity (ρ_e) of the A-MCA material compared with other SMMs and MCAs.

The investigated A-MCA material is compared with pure metals, Fe–Si alloys[3], Fe–Co alloys[4], Fe–Ni alloys[5] and other established MCAs[6-9]. ρ_e of the new A-MCA material is higher than that of all pure metals, Fe–Si alloys, Fe–Co alloys, Fe–70~80Ni alloys. It is comparable to that of Fe–40~50Ni alloys and recently-established MCAs.

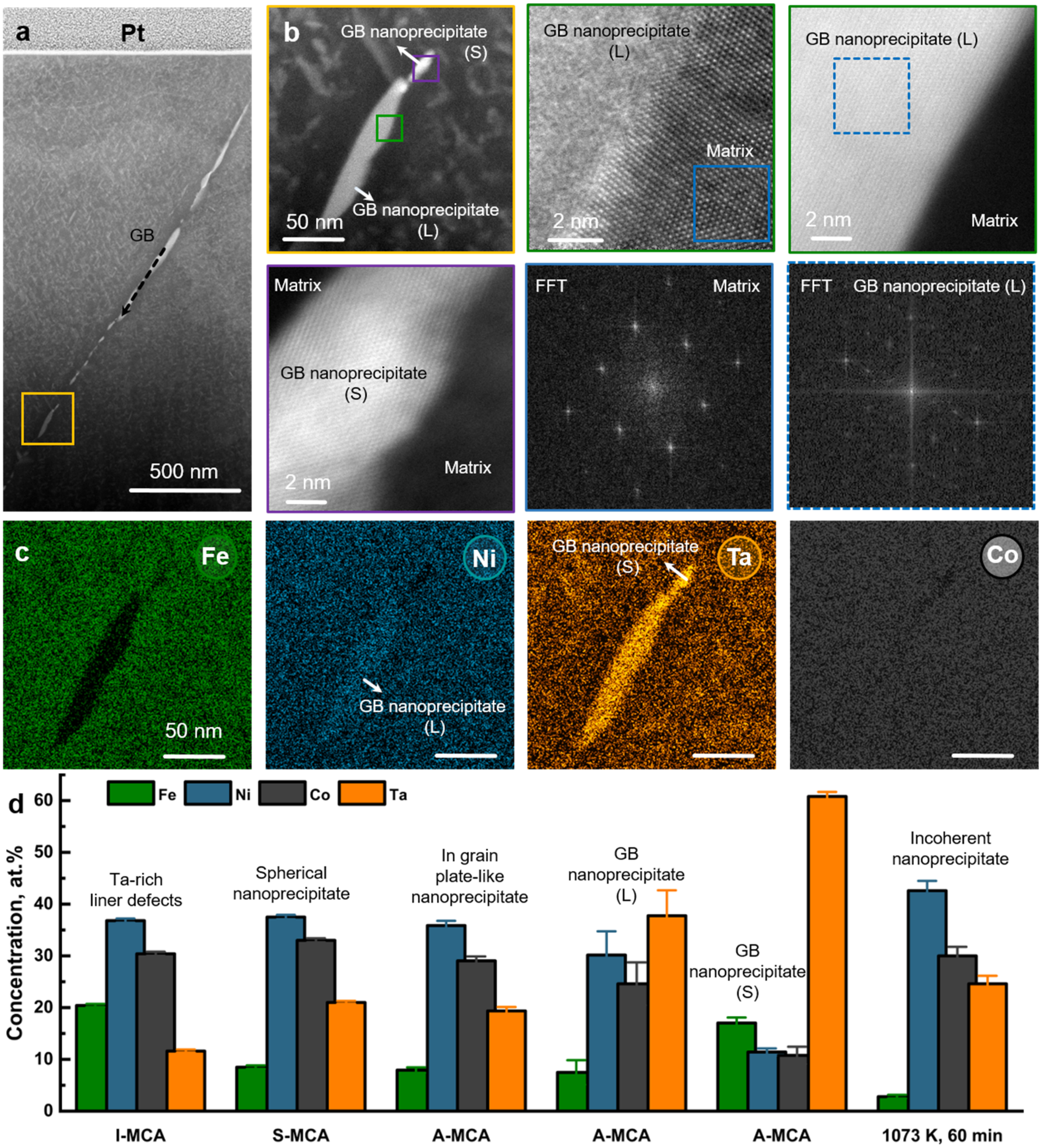

Supplementary Figure 15. STEM HAADF images and STEM EDS analysis of the microstructures and chemical compositions of precipitates with different morphologies in the A-MCA material.

a Overview STEM micrograph showing discontinuous heterogeneous precipitates at a grain boundary (GB). **b** Enlarged view of the region marked by an orange frame in (**a**) showing two types of heterogeneous precipitate at a GB. The corresponding atomically-resolved STEM

HAADF image (green frame, large precipitate; purple frame, small precipitate) and FFT patterns (solid blue frame, matrix; dashed blue frame, large precipitate) indicate that both precipitates are incoherent with the matrix. **c** STEM EDS elemental maps of the region marked by an orange frame in (**a**). **d** Comparison of average chemical compositions of microstructural defects for different thermomechanical processing sequences. L, large; S, small.

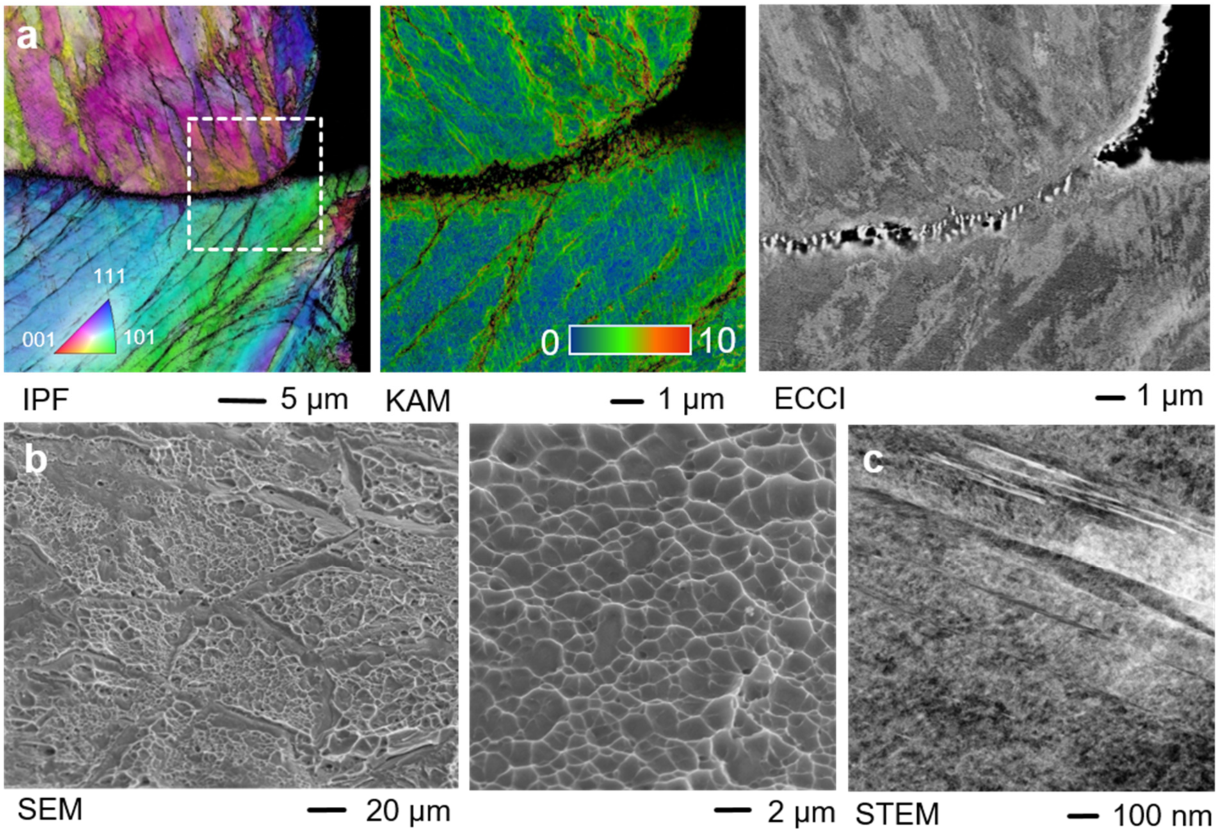

Supplementary Figure 16. Tensile fracture microstructure analysis of the A-MCA material. **a** Cross-sectional correlative EBSD-ECCI analysis. The enlarged area from the IPF map (left) shows the distribution of KAM values near the fracture (middle). Compared to grain interiors with relatively lower KAM values (~1°), heterogeneous precipitates at the GB show higher KAM values (~5°). ECC imaging (right) shows detachment of heterogeneous precipitates at the GB. **b** SEM analysis showing fractography. The low magnification image of the fracture surface (left) reveals different fracture mechanisms: cleavage fracture at the GB and ductile fracture in grains characterized by nano-scaled dimples in the enlarged view (right). **c** Representative TEM image of the cross-sectional deformation microstructure near the fracture showing multiple parallel mechanical twins.

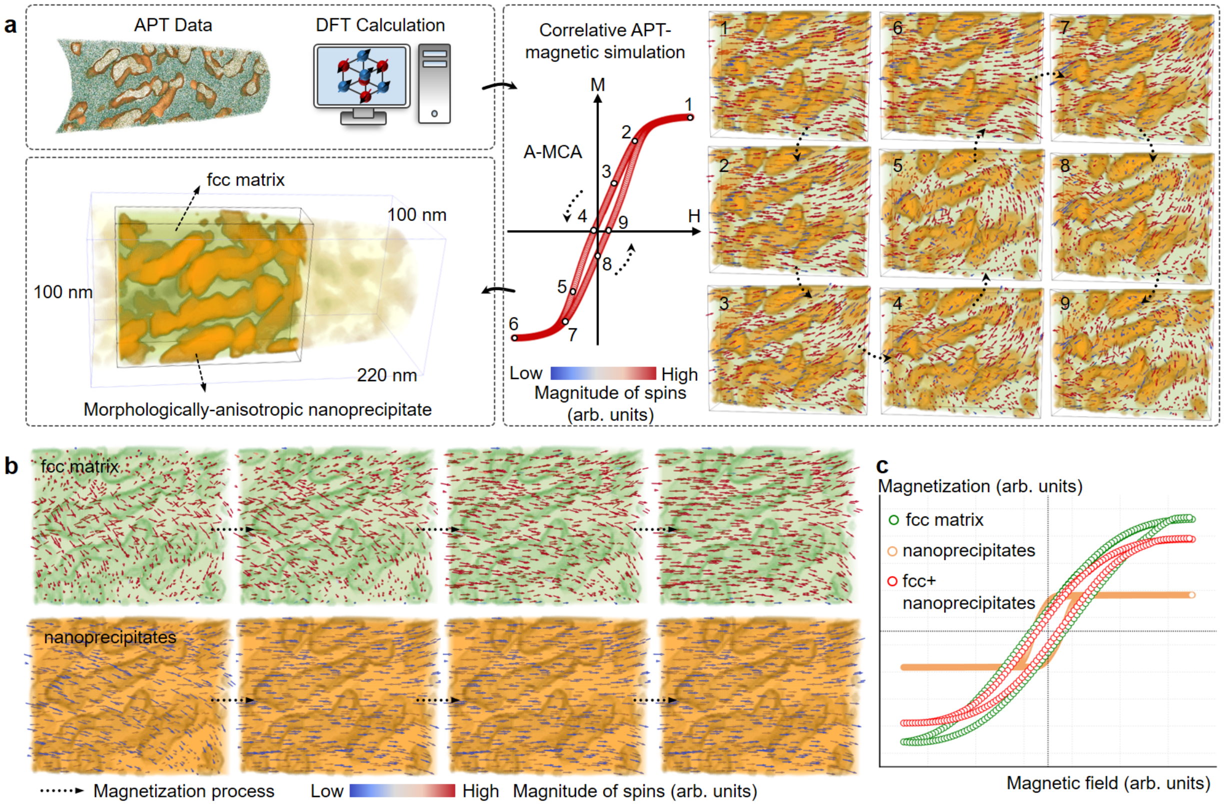

Supplementary Figure 17. Procedure for analyzing 3D magnetic simulations of plate-like precipitates using a machine-learning-inspired method.

a First, experimentally-measured APT data and computationally-calculated parameters were used to simulate the 3D magnetic configuration. A boundary detection algorithm was used to determine the phase boundaries by leveraging the concept of convolutional layers[10]. Second, the compositions and structures of the individual phases, including the spatial connectivity of local variations, were used to simulate the effect of exchange interaction between the strong ferromagnetic matrix precipitates. Third, the area of interest was subtracted for further investigation and visualization[11]. **b** Effect of exchange interaction of the pure fcc matrix (top images) and precipitates (bottom images), respectively. **c** Hysteresis loop of the fcc matrix, precipitate and P-MCA (matrix+precipitate) obtained micromagnetic simulation. The coercivity of the P-MCA is smaller than that of the pure fcc matrix and precipitate.

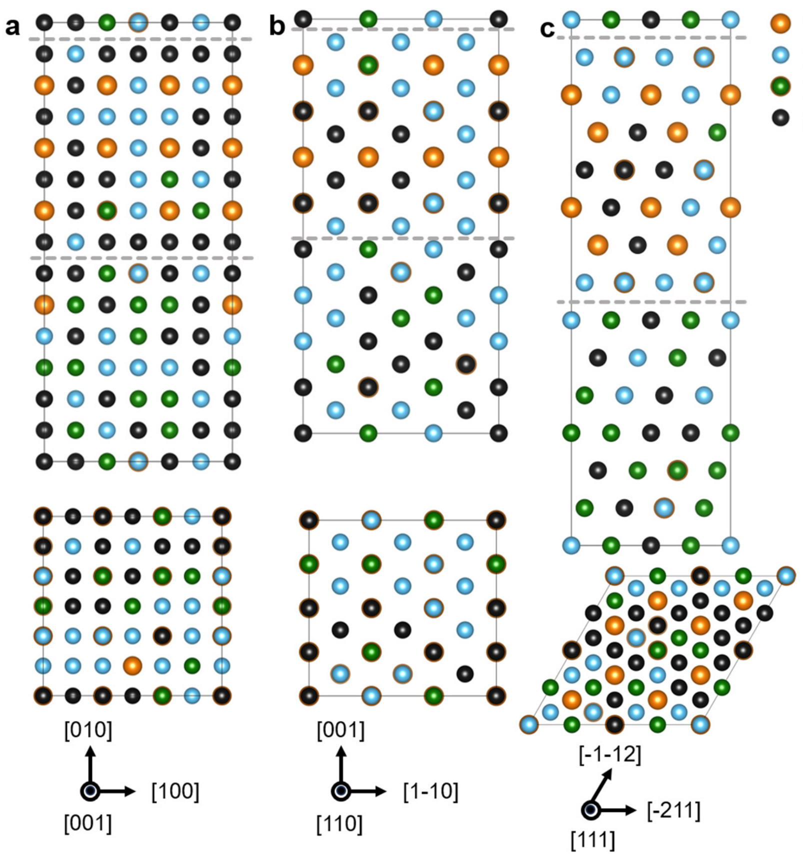

Supplementary Figure 18. Crystal structures of the interface between the matrix and precipitate in the A-MCA.

Side views (top) and top views (bottom) of interface structures. **a** $fcc_{(100)}/L1_{2(100)}$, **b** $fcc_{(110)}/L1_{2(110)}$, and **c** $fcc_{(111)}/L1_{2(111)}$.

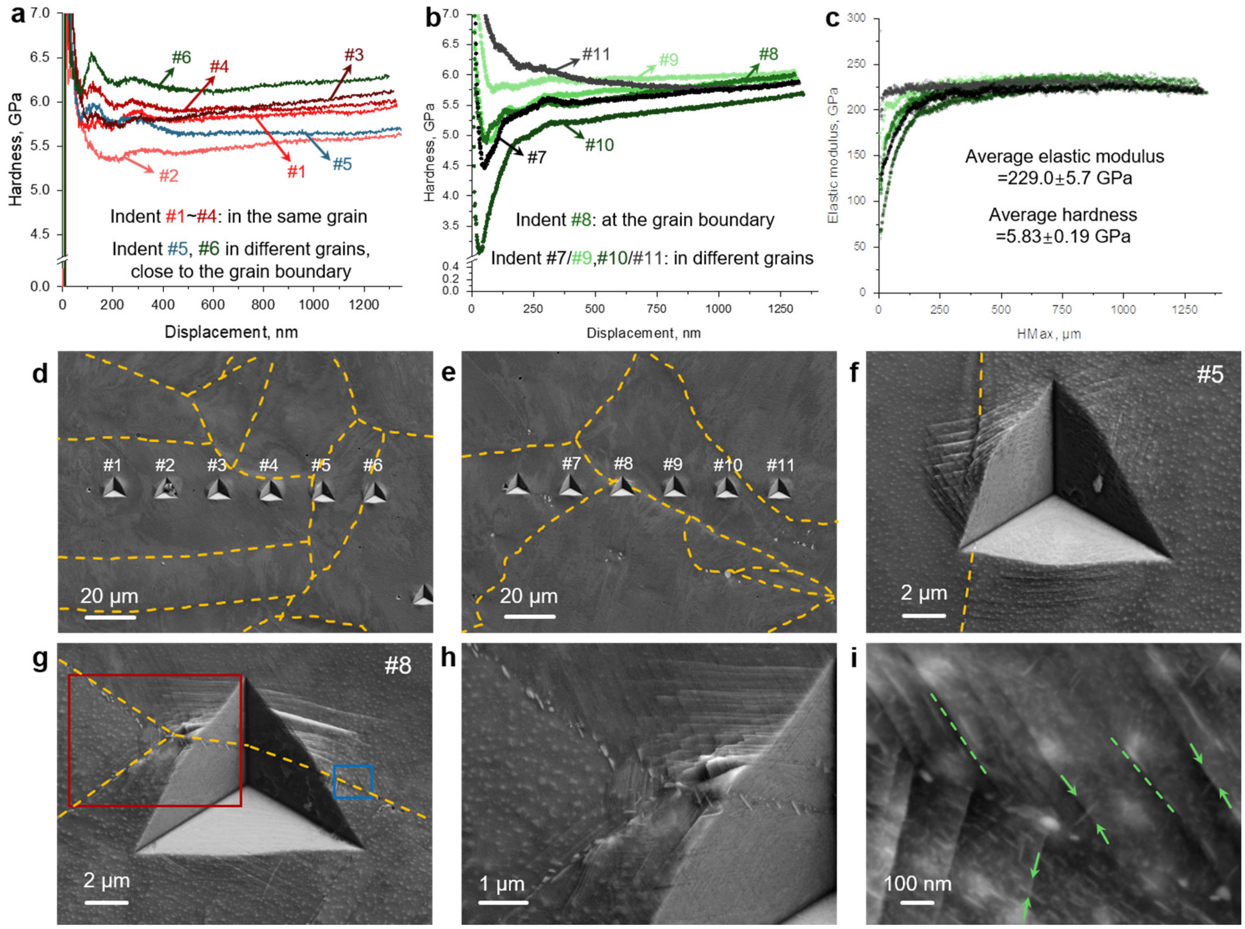

Supplementary Figure 19. Nanoindentation analysis of the A-MCA material.

The influence of crystallographic orientation and of the presence of a GB on hardness were investigated using a nanoindentation measurement. **a** Hardness-displacement curves of indents distributed in one grain (#1~#4) and close to a GB (#5, #6). **b** Hardness-displacement curves recorded from indents distributed in different grains (#7, #9 and #10, #11) and at a GB (#8). For better interpretation and comparison, averaged hardness values obtained from 18 measurements for indent depths of 500~1200 nm were converted to flow strength by applying the Tabor relation[11] as 1.94±0.06 GPa. The stress is comparable to the value at 8% engineering strain achieved by the tensile test (2.04 GPa). **c** Elastic modulus-displacement behaviour. No significant orientation dependence on elastic modulus and hardness was observed. **d**, **e** SEM characterization showing the overall microstructure of the indents after testing. Orange dashed lines, GB. **f** SEM analysis of one indent near the GB. Grain boundary migration and reorientation of slip lines across the GB are observed. **h** Enlarged view of the region marked by a red frame in (**g**), showing slip lines near

a triple GB. **i** Enlarged view recorded from the region marked by a blue frame in (**g**), showing shearing of plate-like precipitates perpendicular to slip lines (green arrows). The green dashed lines show slip lines parallel to the longitudinal edge of the plate-like precipitates.

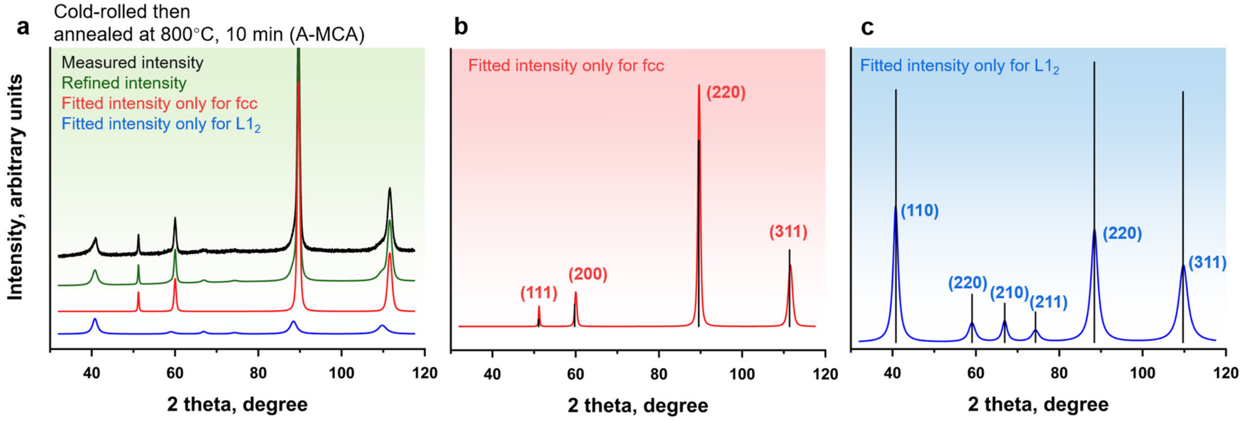

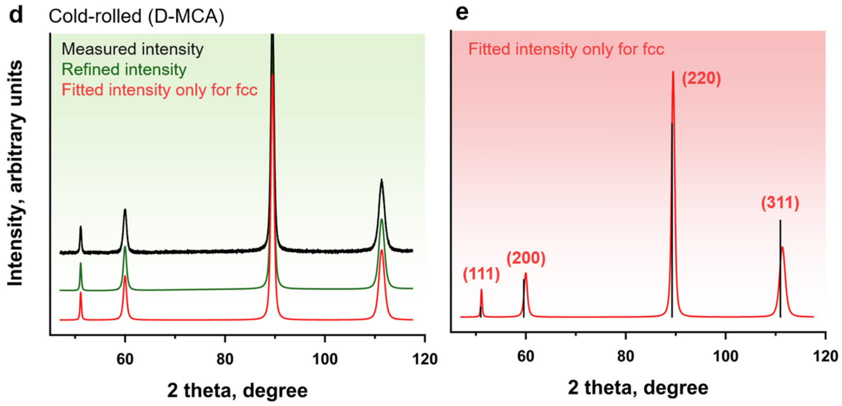

f

fcc	A-MCA
{111}	3.58728±0.000120 Å
{002}	3.57923±0.000098 Å
{022}	3.59135±0.000034 Å
{311}	3.59000±0.000043 Å

fcc	D-MCA
{111}	3.5885±0.00013 Å
{002}	3.5778±0.00013 Å
{022}	3.60077±0.000079 Å
{311}	3.5939±0.0001 Å

Supplementary Figure 20. Refined XRD analysis for the A-MCA and D-MCA materials.
a Measured and refined XRD analysis for the A-MCA material. **b**, **c** Corresponding fitted intensity and peak positions only for the fcc and L1$_2$ phases, respectively. **d** Measured and refined XRD analysis for the D-MCA material. **e** Corresponding fitted intensity and peak positions for the fcc phase. **f** Fitted lattice parameters for the fcc phase in the D-MCA and A-MCA material. The fitting results show that potential internal stress is present in the current MCAs.

Supplementary Tables

Supplementary Table 1. Equilibrium lattice parameters calculated using density functional theory (DFT).

	C_{11}	C_{12}	C_{44}	SFE	M_S	K_1	A_{ex}
		(GPa)		(mJ·m^{-2})	(MA·m^{-1})	(J·m^{-3})	(pJ·m^{-1})
fcc	211	165	287	73.9	161.1	964	26.5
$L1_2$	284	176	377	/	38.7	453	7.8

Supplementary Table 2. Sample identification, microstructural features, and thermomechanical processing of the investigated $Fe_{35}Co_{30}Ni_{30}Ta_5$ (at.%) material. "HR", "CR", and "WQ" refer to "hot-rolling", "cold-rolling", and "water quenching", respectively.

Sample identification	Thermomechanical processing	Microstructural features
B-MCA	Cast+HR+homogenization+WQ	Equiaxed grains
S-MCA	Cast+HR+homogenization+WQ+ annealing@1073 K for 10 min+WQ	Equiaxed grains +coherent disordered spherical precipitate
D-MCA	Cast+HR+homogenization +WQ+CR	Elongated grains+texture
A-MCA	Cast+HR+homogenization+WQ+ CR+annealing@1073 K for 10 min+WQ	Elongated grains+texture+coherent ordered plate-like precipitate
I-MCA	Cast+HR+homogenization+WQ+CR+ annealing@973 K for 10 min+WQ	Elongated grains+texture+chemically decorated dislocations
1073 K, 60 min-MCA	Cast+HR+homogenization+WQ+ CR+annealing@1073 K for 60 min+WQ	Elongated grains+texture+ incoherent ordered plate-like precipitate

Supplementary Table 3. Mechanical properties of the current MCAs with different thermomechanical processing conditions.

Alloy	Yield strength (σ_y), GPa	Ultimate tensile strength (σ_{uts}), GPa	Fracture to elongation (ε_f), %
B-MCA	0.50	0.90	53.4
S-MCA	0.87	1.25	24.8
D-MCA	1.32	1.41	19.5
A-MCA	1.93	2.04	12.6

References

[1] Bunge, H. J. Orientation of Individual Crystallites. in Texture Analysis in Materials Science (Elsevier, 2013).

[2] Jang, T. J. et al. Shear band-driven precipitate dispersion for ultrastrong ductile medium-entropy alloys. *Nat Commun* **12**, 4703 (2021).

[3] Chih-Wen Chen. Magnetism and Metallurgy of Soft Magnetic Materials. (Courier Corporation, 2013).

[4] Sundar, R. S. & Deevi, S. C. Soft magnetic FeCo alloys: alloy development, processing, and properties. *Int. Mater. Rev.* **50**, 157-192 (2005).

[5] Rösler, J., Harders, H. & Bäker, M. Mechanical Behaviour of Engineering Materials: Metals, Ceramics, Polymers, and Composites. (Springer Science & Business Media, 2007).

[6] Zhou, K. X. et al. FeCoNiAlSi high entropy alloys with exceptional fundamental and application-oriented magnetism. *Intermetallics* **122**, 106801 (2020).

[7] Li, P., Wang, A. & Liu, C. T. Composition dependence of structure, physical and mechanical properties of $FeCoNi(MnAl)_x$ high entropy alloys. *Intermetallics* **87**, 21–26 (2017).

[8] Zhao, C. et al. Effect of strong magnetic field on the microstructure and mechanical-magnetic properties of AlCoCrFeNi high-entropy alloy. *J Alloys Compd* **820**, 153407 (2020).

[9] Zuo, T. T., Li, R. B., Ren, X. J. & Zhang, Y. Effects of Al and Si addition on the structure and properties of CoFeNi equal atomic ratio alloy. *J Magn Magn Mater* **371**, 60–68 (2014).

[10] Vansteenkiste, A. et al. The design and verification of MuMax3. *AIP Adv* **4**, 107133 (2014).

[11] Zhang, P., Li, S. X. & Zhang, Z. F. General relationship between strength and hardness. *Materials Science and Engineering: A* **529**, 62–73 (2011).
